# The physiological correlates of interpersonal space

**DOI:** 10.1038/s41598-021-82223-2

**Published:** 2021-01-28

**Authors:** Michela Candini, Simone Battaglia, Mariagrazia Benassi, Giuseppe di Pellegrino, Francesca Frassinetti

**Affiliations:** 1 Department of Psychology, University of Bologna, Viale Berti Pichat, 5, 40127 Bologna, Italy; 2 Istituti Clinici Scientifici Maugeri IRCCS, Operative Unit for Recovery and Functional Rehabilitation of the Institute of Castel Goffredo, 46042 Mantova, Italy; 3 CsrNC, Center for Studies and Research in Cognitive Neuroscience, 47521 Cesena, Italy

**Keywords:** Neuroscience, Cognitive neuroscience

## Abstract

Interpersonal space (IPS) is the area around the body that individuals maintain between themselves and others during social interactions. When others violate our IPS, feeling of discomfort rise up, urging us to move farther away and reinstate an appropriate interpersonal distance. Previous studies showed that when individuals are exposed to closeness of an unknown person (a confederate), the skin conductance response (SCR) increases. However, if the SCR is modulated according to participant’s preferred IPS is still an open question. To test this hypothesis, we recorded the SCR in healthy participants when a confederate stood in front of them at various distances simulating either an approach or withdrawal movement (Experiment 1). Then, the comfort-distance task was adopted to measure IPS: participants stop the confederate, who moved either toward or away from them, when they felt comfortable with other’s proximity (Experiment 2). We found higher SCR when the confederate stood closer to participants simulating an IPS intrusion, compared to when the confederate moved farther away. Crucially, we provide the first evidence that SCR, acting as a warning signal, contributes to interpersonal distance preference suggesting a functional link between behavioral components of IPS regulation and the underlying physiological processes.

## Introduction

The interpersonal space (IPS) refers to the area around one’s own body, into which social interactions typically occur^1^. When other individuals infringe on our IPS, feelings of discomfort and even fear may rise up^2,3^, urging us to move farther away and promptly reinstate a safety and appropriate interpersonal distance^4–11^.

In this respect, a key issue concerns the potential mechanisms subtending IPS regulation. One of these mechanisms could be the ability to anticipate aversive events during social interactions, in order to mitigate the effect of possible threat. In line with this, the amygdala, a brain structure that is crucially implicated in the detection of threatening situations^12–14^, is selectively activated by proximal social stimuli^15,16^. For example, in an fMRI study, Kennedy and colleagues^15^ found that the activity in the amygdala is enhanced when an individual is standing close-by to the participant, as compared to when that individual is far away. Furthermore, the authors described a patient with bilateral amygdala damage who exhibited an abnormally small IPS compared to a group of neurologically healthy participants. Given the pivotal role played by the amygdala in social approach and avoidance behaviours, both in primates and humans^17–21^, it is possible to argue that this brain region is necessary for eliciting emotional responses to proximal social stimuli, and thus regulating a safety IPS boundary between self and others.

Potentially threatening situations, such as others’ proximity, triggers a number of physiological reactions which are considered indicators of the level of arousal and distress. For instance, a study on public transport passengers showed that participants, exposed to closeness of strangers, increased their salivary cortisol as well as reported high level of distress^22^. More recently, a link was found between the respiratory sinus arrhythmia, an index of autonomic nervous system activity, and the perception of threating others located close to the participant’s body^23^. Another widely used measure of the autonomic nervous system activity is the skin conductance response (SCR). McBride et al.^24^ found that SCR was greater when an unfamiliar person was close to, relative to far from, the participant’s body. Moreover, participants’ physiological response was sensitive to the direction of approaching movement, with greater SCR when an unknown person approached them from the front, compared to rear space.

So far, extant studies demonstrated that the others’ intrusion into one's own IPS elicits emotional reactions in individuals which could be recorded at the physiological level. However, none of the previous studies combined physiological responses to social proximity with a subjective measure of IPS estimated in an ecological task, i.e. the space that we prefer to maintain from others.

The aim of the present study is twofold: to investigate the relationship between social proximity and autonomic responses in an ecological context (Experiment 1), and to verify whether physiological individual responses change according to preferred IPS (Experiment 2).

**Figure 1:**
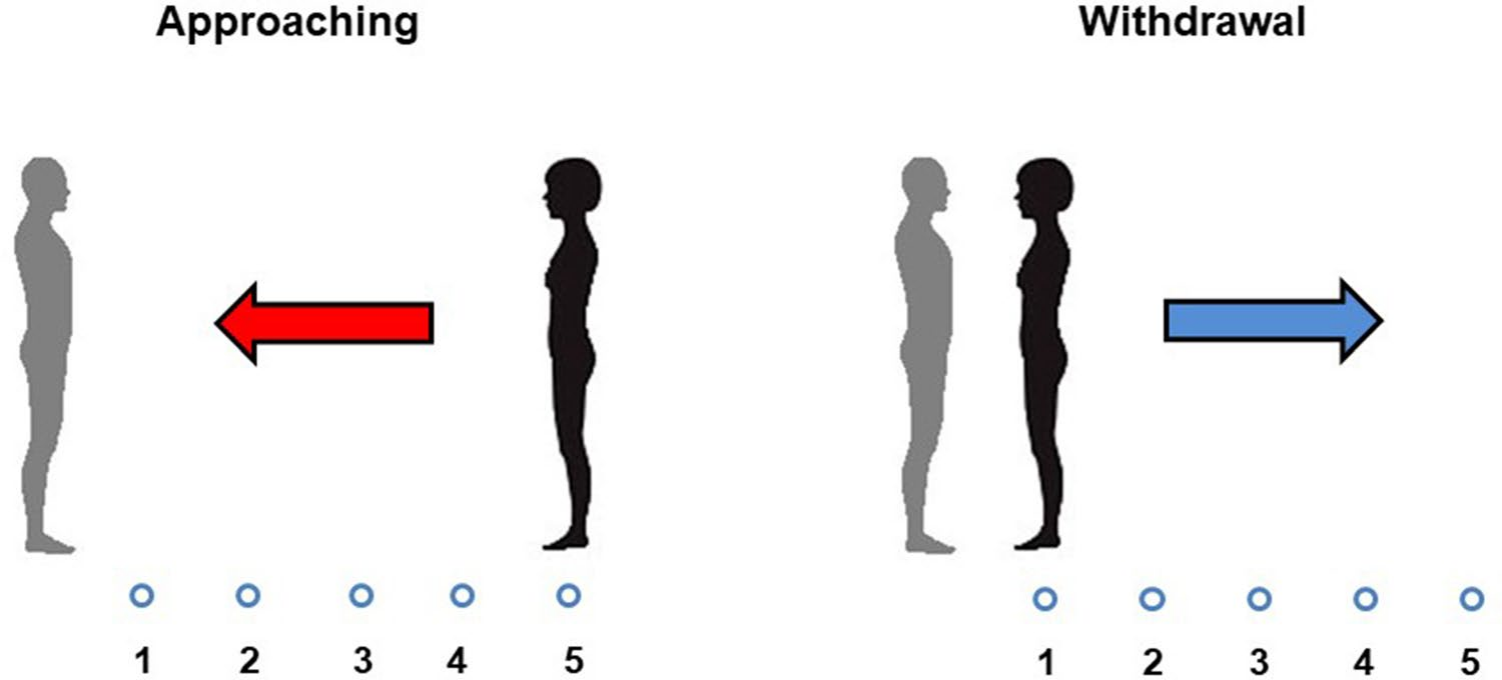
Schematic representation of the experimental procedure. The participant (grey) and the confederate (black) were depicted. Arrows indicate the confederate movement direction: toward the participant (approaching movement; left panel) or away from the participant (withdrawal movement; right panel) according to five different spatial positions (1–5).

To address these questions, we conducted two experiments in which an unknown person (confederate) stood in front of the participant at a close (25 cm) or far (105 cm) starting position. In Experiment 1, participant’ SCR was recorded when confederate moved and briefly stopped, at five different *spatial positions* (25–45–65–85–105 cm; see Fig. 1), *approaching* to or *withdrawing* from the participant. In Experiment 2, the confederate slowly and continuously *approached* to or *withdrew* from participant who stopped the confederate at a comfortable distance. Thus, to attribute a specific physiological response to a specific distance, SCR was measured only in Experiment 1 in which confederate moved and stopped at predefined spatial positions. In Experiment 2, to measure preferred interpersonal distance, participants performed a classical comfort-distance task in which confederate moved in a continuous way^5,9–11,25–29^.

We expect that physiological responses, as assessed by SCR, should increase according to spatial closeness to an unfamiliar adult. In particular, the increment of SCR should emerge in the approaching condition when the confederate simulates an IPS intrusion, compared to the withdrawal condition, when the confederate moves farther away from the participant. Furthermore, we also expect that SCR is differently modulated according to the participant’s preferred IPS, as measured in the comfort-distance task. Finally, previous studies documented that individual characteristics, such as gender, influence the preferred interpersonal distance: female participants preferred larger IPS as compared to male participants^6,8,25,30,31^. Here we explore whether this effect is present also at physiological level resulting in a modulation of SCR. Together, these results would provide the first physiological evidence concerning IPS regulation in a naturalistic and ecological social environment.

## Results of Experiment 1

### A physiological measure of interpersonal distance

To study the physiological correlates of spatial proximity, SCR (baseline corrected peak-to-peak, see “Material and methods” for further details) was analyzed by using a Generalized Linear Mixed Model with Movement (Approaching vs. Withdrawal) as within-subject factor, and Spatial position (1–5) as covariate. The participants’ Gender (female and male), Spatial position and Movement variables were entered as fixed effects.

**Figure 2:**
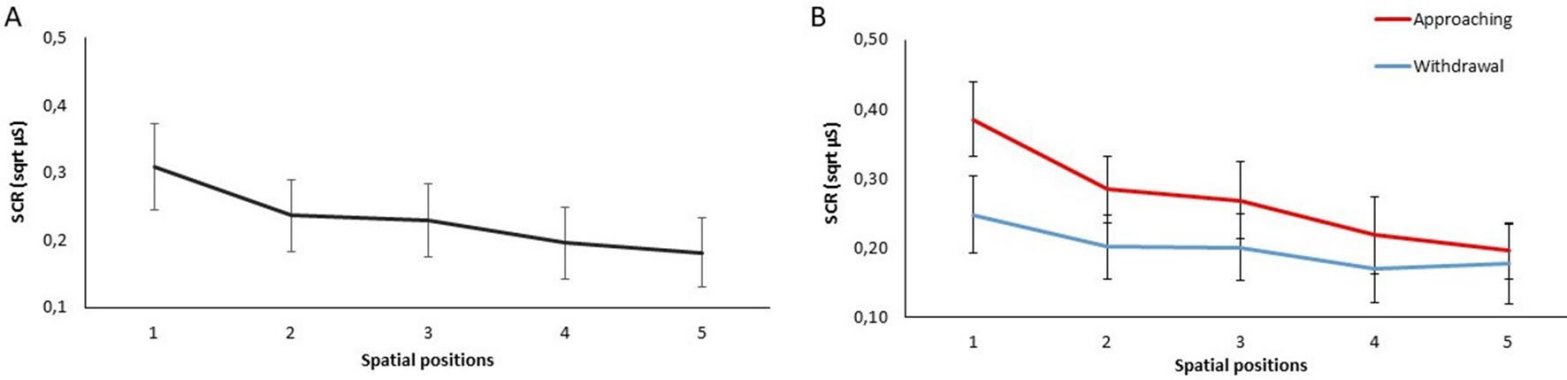
A physiological measure of interpersonal distance. The graph illustrates modulations of skin conductance responses (SCRs; expressed in sqrt µS). In the left panel, SCR is expressed as a function of Spatial positions (1–5) (**A**). In the right panel, SCR is expressed as a function of Movement (Approaching and Withdrawal) and Spatial positions (1–5) (**B**). Error bars indicate 95% confidence intervals.

The analysis revealed a significant effect of **Movement** [F(1,225) = 32.224; p = 0.0001] on SCR: higher SCR was found when the confederate moved toward the participant (approaching = 0.27 sqrt µS; 95% CI = [0.33, 0.21]) compared to when the confederate moved farther away (withdrawal = 0.20 sqrt µS; 95% CI = [0.24, 0.14]). Moreover, the variable **Spatial position** [F(1,225) = 36.483; p = 0.0001] was significant: the close was the confederate to the participant, the higher was the SCR (coefficient b = − 0.001; see Fig. 2A). Furthermore, we found a significant interaction **Movement × Spatial position** [F(1,225) = 12.870; p = 0.001]. Interestingly, in the approaching condition, the SCR rapidly increased according to the spatial positions progressively occupied (from position 5 to 1) by the confederate as compared to the withdrawal condition (coefficient b = − 0.001; see Fig. 2B). Furthermore, estimated means and CI in Fig. 2B shows that the greater difference between approaching and withdrawal conditions clearly emerged when the confederate occupied the 1 and 2 positions, i.e., the positions closer to participants body. The variable **Gender** [F(1,225) = 0.190; p = 0.663] was not significant.

### Correlation between participants’ anxiety score and the SCR

We perform two Pearson’s correlations between participants’ anxiety score and the SCR (i.e., the mean SCR obtained when the spatial positions were collapsed), separately for approaching and withdrawal conditions. The analysis failed to reveal significant results both when the state anxiety score (approaching: t = 0.112; p = 0.61; withdrawal: t = 0.09; p = 0.68) and trait anxiety score were considered (approaching: t = − 0.03; p = 0.89; withdrawal: t = 0.66; p = 0.77).

## Results of Experiment 2

### A measure of interpersonal distance

To measure the IPS, the preferred distances judged by participants (expressed in cm) were analyzed by means of ANOVA considering the variables Movement (Approaching and Withdrawal) as within-subject factors, and participant’s Gender (female and male) as between subject-factor. The ANOVA failed to reveal a significant effect both for the variable **Movement** [F(1,21) = 0.38; p = 0.543; η^2^_p_ = 0.02] (Approaching = 51.3 cm; Withdrawal = 52.4 cm), as well as for the participant’s **Gender** [F(1,21) = 0.06; p = 0.804; η^2^_p_ = 0.01] (female = 52.3 cm; male = 51.1 cm). The interaction **Movement × Gender** was not significant [F(1,21) = 0.001; p = 0.99; η^2^_p_ = 0.001]. It is worth noting that the estimated IPS, that is the distance at which participant felt comfortable and stopped the confederate, was around 50 cm as well as the distance (position 2) at which the SCR increased in the previous task.

### Correlation between participants’ anxiety score and the preferred interpersonal distance

We perform two Pearson’s correlations between participants’ anxiety score and the mean preferred interpersonal distance, separately for approaching and withdrawal conditions. The analysis failed to reveal significant results both when the state anxiety score (approaching: t = 0.24; p = 0.27; withdrawal: t = 0.27; p = 0.21) and trait anxiety score were considered (approaching: t = 0.22; p = 0.305; withdrawal: t = 0.33; p = 0.13).

### Relationship between preferred interpersonal distance and physiological measure

**Figure 3:**
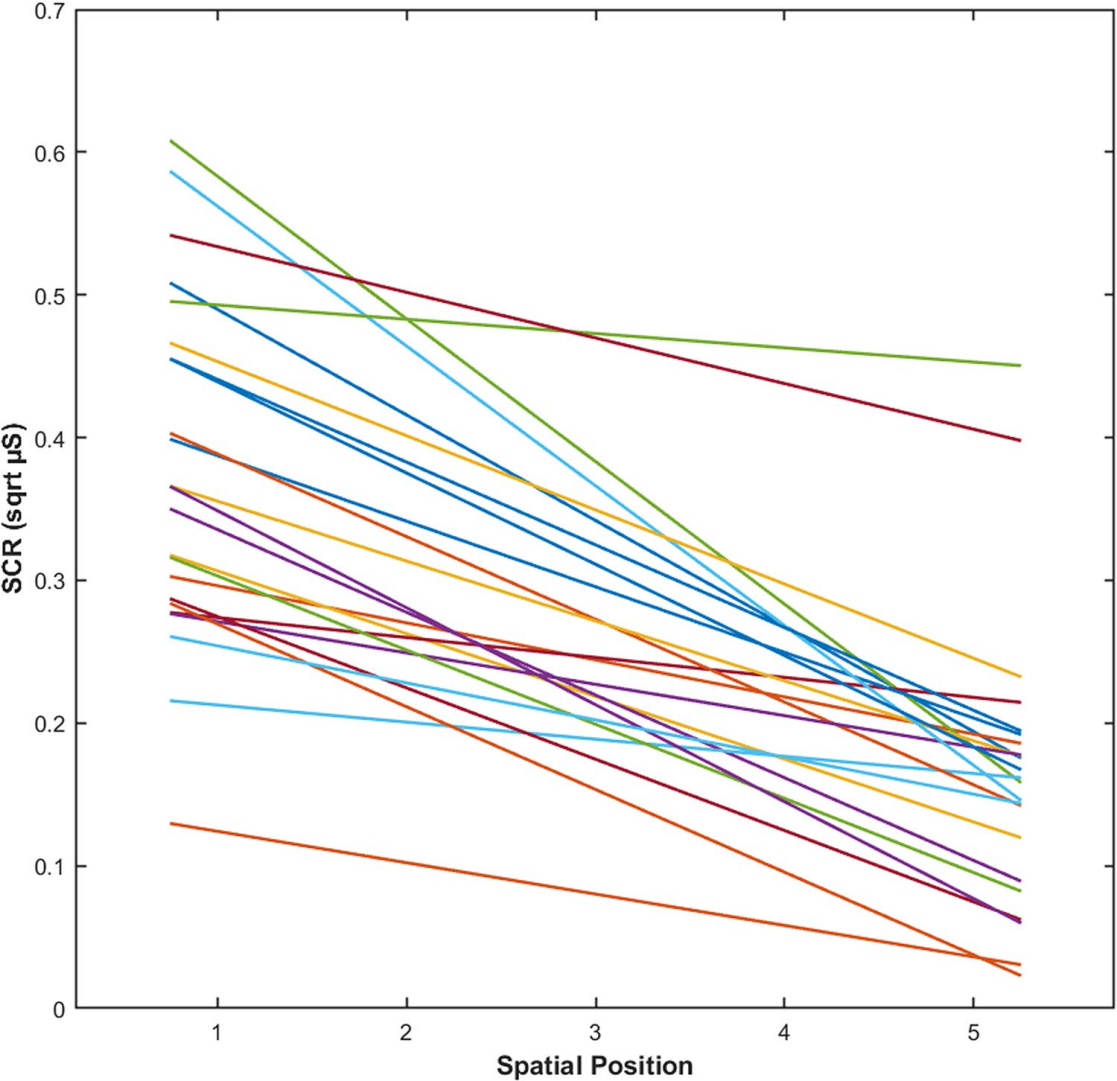
Relationship between preferred interpersonal distance and physiological measure. The graph depicts the best-fitting regression lines showing the relationship between SCR and spatial position in the approach condition of Experiment 1 for each individual participant (N = 23). The slope of each line, indicating the rate at which SCR changed as the confederate approached the participant, were correlated with participants’ preferred interpersonal distance, as assessed in the comfort-distance task of Experiment 2.

Crucially for the aim of the study, we found a significant correlation between individual preferred interpersonal distance and slope parameters of the SCR functions [F(1,21) = 5.07 p = 0.0351, with an R adjusted of 0.19], indicating that the higher (i.e., steeper) the slope, the larger was the preferred interpersonal distance (SE = 0.18, β = -0.44, t = − 2.25; p = 0.035; see Fig. 3). This finding suggests that interpersonal distance preferences change accordingly to individual physiological reactions to the physical proximity of another person.

## Discussion

In recent years, a growing body of evidence has contributed to clarify the mechanisms involved in the ability to regulate the interpersonal distance between individuals. Previous findings coming from social psychology and cognitive neuroscience have been mainly focused on the key role played by high-level factors, such as the social perception of others (i.e., the gender, the age or the level of affiliation between individuals), and their top-down influence on the preferred IPS^6,25,28,30,32,33^. Nonetheless, it is largely unexplored how autonomic signals coming from one’s own body contribute to define social proximity more or less uncomfortable, and thus influencing the IPS. In this respect, there are very few studies which report an increased arousal response to the physical proximity of an unfamiliar person^22,24,34,35^. However, none of these studies examined whether physiological arousal was indeed related to preferred interpersonal distance.

To address this issue, we combined, for the first time, a physiological measure of social proximity with a behavioral measure of IPS estimates in a real social context. To this aim, participants’ SCR was recorded when they faced an unfamiliar person (confederate) at different distances; then, the relationship between SCR and preferred IPS, measured with a classical comfort-distance task^5,9–11,26–29^, was analyzed.

We observed a modulation of SCR depending on confederate’s spatial position. Indeed, SCR rapidly increased from far (position 5–105 cm) to close distance (position 1–25 cm). This result could be accounted for by a feeling of discomfort due to the proximity of an unfamiliar adult close to one’s own body^22,24^. Indeed, when individuals are exposed to closeness of an unknown person, regardless of whether it induces a discomfort or a pleasant feeling, this behavior is arousing because it may be perceived as socially non-appropriate. One interpretation is that the closer an unknown individual is to one’s own body, the more likely it is perceived as uncomfortable, and stronger is the elicited defensive response^36^ and the SCR increases.

Significantly, in the present study the increment of SCR was modulated by the type of movement: in the approaching condition, SCR gradually increased as the confederate was getting near, whereas in the withdrawal condition the modulation of SCR, as a function of the spatial position progressively occupied by the confederate, failed to emerge. This difference is particularly evident at distances close to participant’s body (position 1 and 2, at 25 and 45 cm, respectively). Interestingly, this result suggests that the different SCR modulation between approaching and withdrawal condition observed at closer distance can be explained, not only by the spatial position per se, but also by the ability to anticipate the subsequent confederate’s movement. Thus, the SCR is influenced by the interaction between two factors (1) the confederate’s closeness to participant, and (2) the social context within which the movement is performed: a potential threat and danger in the approaching condition, and a harmless and safe situation in the withdrawal condition. To explain this result, we hypothesize that the expectation of being touched by a unknown person can elicit the participant’s autonomic reactivity. In keeping with this finding, recent fMRI studies showed higher activation of participants’ somatosensory cortex when the pictures of approaching, rather than withdrawing, faces were presented^20,37–39^. Thus, the activation of somatosensory cortex is mainly related to the expectation of a physical contact of another person^20,39^.

Crucially, the correlation analysis demonstrates a relationship between preferred interpersonal distance and individual physiological response, suggesting that SCR depends not only on the spatial distance between participant and confederate, but it is also functionally linked to IPS preferred by participants. One possible interpretation is the following: lower is the level of tolerance for physical proximity of an unknown person, higher is the level of arousal induced by others’ proximity.

Finally, since previous studies documented that individual characteristics, such as gender, influence the IPS, here we explored whether this effect is present also at physiological level resulting in a modulation of SCR. We did not find an effect of participants’ gender both at physiological level, in Experiment 1, and at behavioral level, in Experiment 2. The lack of this effect may be accounted for by the experimental setting adopted, especially for the type of dyads we contrasted. Indeed, previous behavioral research showed that female-female dyads preferred shorter IPS as compared to male-male dyads, and mixed male–female dyads were in the middle, but did not differ significantly from either of the other dyads^40^. However, we compared female-female and mixed dyads and this may explain the lack of significant effect for the variable gender we reported.

To sum up, the novelty of our findings is that the physiological response elicited by proximity to another person may play a crucial role on how we regulate the distance between ourselves and others during social interaction. This is an interesting point that highlights how autonomic response, which we often fail to detect consciously^41,42^, may nevertheless influence interpersonal space boundary^4,15,43^, thus offering a new perspective on the implicit component of social proximity.

These results may have important implications in developing both theoretical framework and new treatments for psychopathological disorders characterized by social disability and an impairment in IPS regulation, such as autism, schizophrenia and social anxiety^3,9–11,15,44–48^.

## Material and methods

### Experiment 1

#### Participants

A total of 23 right-handed healthy young adults participated in the study (13 females; mean age = 24.95 years, SD 3.84 years; age range 20–35 years; mean education = 17.17 years, SD 1.11 years). A priori power analysis was conducted to estimate the sample size by using G*Power. For a within-group ANOVA, we obtained that the required sample was 21 participants with alpha = 0.05, power = 0.80, correlation among repeated measure = 0.7 and f = 0.25). A medium effect size (η^2^_p_ = 0.25) was specified based on a previous study conducted in our laboratory^27^. Participants were students from University of Bologna and were recruited through campus advertisements according with the following criteria. Participants were screened to ensure that they had no history of neurological and psychiatric diseases and self-reported normal or corrected-to-normal vision. None of the participants were taking any medication affecting the central nervous system at the time of the experimentation. Since, it is known that anxiety may affect autonomic responses^3,49^, the level of anxiety was measured by means of the State-Trait Anxiety Inventory (STAI-Y)^50^, a self-report measure of state and trait anxiety. None of participants reported pathological level of state (cut-off: 58.1; mean score = 38.43; SD 8.03; range 37.95 ± 10.09) or trait anxiety (cut-off: 58.8; mean score = 42.48; SD 8.76; range 39.63 ± 9.63) and all scores fall within the range of normality [for the Italian adaptation see^51^].

The study was conducted in accordance with the ethical principles of the World Medical Association Declaration of Helsinki and was approved by the Ethics Committee of the Department of Psychology of the University of Bologna. All participants were naive as to the purpose of the study and provided informed written consent to participation after being informed about the procedure of the study.

#### Physiological recording and data processing

Skin conductance responses were recorded with a Biopac MP-150 at 200 Hz sampling rate (BIOPAC Systems Inc., Goleta, California, USA) and collected with AcqKnowledge 3.9 software (BIOPAC Systems) for offline analysis. Signal was acquired with two Ag/AgCl electrodes (TSD203; BIOPAC Systems), filled with isotonic hyposaturated conductant gel, and attached to the distal phalanges of the second and third finger of the participant left hand. A Biopac EDA100C (BIOPAC Systems) was used as measurement instrument of SCR (gain switch set to 5 μS/V, low pass to 35 Hz, high pass to DC). SCR collected data were analyzed offline using MATLAB environment (Version R2018a; MathWorks Inc., Natick, Massachusetts, USA).

Each trial was extracted from the entire SCR signal and to reduce inter-individual variability, a baseline correction was conducted using the mean value of the 1 s before each presentation of the stimulus as a baseline^52–54^. Then, for each baseline corrected trial, peak-to-peak value was calculated as the amplitude of the largest deflection during the 0.5–4.5 s time window, after trial onset. The minimum response criterion was 0.02, and smaller responses were encoded as zero. Then, SCR peak-to-peak values were square-root transformed (sqrt) to normalize distribution^55^.

#### Experimental procedure

At the beginning of each trial, participants stood with their arms extended along their trunk in a comfortable and predefined position, leaning against the wall. Before starting, the experimenter asked participants to remain as quiet and still as possible during the SCR recording. Participants were required to maintain their eyes closed while an unfamiliar person (confederate) silently entered in the room. When an acoustic cue was given, participants opened their eyes and saw a female confederate with a neutral facial expression in front of them. They had to maintain eye contact with the confederate until a different acoustic cue was given. The confederate was instructed to carefully check that the eye contact with the participant was maintained for the whole trial duration. Indeed, as previously found in proxemics literature, a time prolonged eye contact with a stranger can induce strong reactions^49,56–58^.

During the experiment, each trial began with the acoustic cue “open” and ended with the acoustic cue “close”, lasting 5 s. Acoustic cues delivering were automatized with a custom-made script in MATLAB as well as the trigger signal in SCR for trials onset. The intertrial interval (ITI) had a variable duration ranging from 9 to 11 s.

The confederate stood facing the participant according to two starting positions: *far* (105 cm) or *close* (25 cm) (see Fig. 1). During the inter-trial interval, participants keep their eyes closed and the confederate get the next spatial position by moving toward or away from the participants, simulating step by step an approach/withdrawal movement. The five spatial positions occupied by the confederate corresponded to the following metric distances: 1 = 25 cm; 2 = 45 cm; 3 = 65 cm; 4 = 85 cm; 5 = 105 cm. When the confederate reached the next spatial position, the acoustic cue was delivered and participants opened their eyes.

The experiment comprised a total of 40 trials in which the conditions (approach/withdraw) were counterbalanced between participants.

#### Statistical analysis

Statistical analyses were performed with SPSS (IBM Statistics for Windows, Version 20.0. Armonk, NY: IBM Corp.) on participants’ SCRs recorded at five spatial positions (1 = 25; 2 = 45; 3 = 65; 4 = 85; 5 = 105 cm). The lack of significant results (p > 0.05) using Kolmogorov–Smirnov test, and skewness and kurtosis values that were not more than twice their standard errors, allowed to consider the dependent variables as normally distributed. Thus, a Generalized Linear Mixed Model (GLMM) was used to investigate differences within experimental phases.

In order to explore the physiological correlates of spatial proximity, SCR was analyzed by using a GLMM with Movement (Approaching vs. Withdrawal) as within-subject factor and Spatial position (1–5) as covariate. The participants’ Gender (female and male), Spatial position and Movement variables were entered as fixed effects. To exclude that potential violations of normality distribution influenced our results, we also adopted a robust estimation of model’ parameters. We indicated the 95% confidence intervals (CI) for all mean values reported.

### Experiment 2

#### Participants

In Experiment 2, participants were the same as in Experiment 1. The Experiment 2 was always conducted after the Experiment 1. All participants performed both experiments in one single session lasting up to 1 h.

#### Experimental procedure

In Experiment 2, the setting was exactly the same as described in the previous section, except that there was no SCR recording during the approach/withdrawal movement.

Testing began with a participant positioned at a predefined location in the room and the confederate standing facing the participant from a far starting position in half of the trails (105 cm), or from a close starting position in the other half (25 cm). The experiment consisted in twelve trials in which a female confederate with a neutral facial expression, slowly approached or moved away from the participant, maintaining the eye contact. The order of the conditions (approach/withdraw) was counterbalanced between participants. Participants were instructed to stop the confederate at their preferred distance (i.e., the distance between themselves and the confederate at which they felt most comfortable). Finally, the chest-to-chest distance at the sternum level was measured with a digital laser meter (Agatec DM100, error ± 0.3 mm/m). All the experimental procedure here described has been conducted in accord with a previous study in which the preferred interpersonal distance was measured by using the comfort-distance task^9^.

#### Statistical analysis

To explore the effects of participants’ gender on the IPS both in approaching and withdrawal condition, the mean distances (expressed in cm) judged by participants during the comfort-distance task in each condition were contrasted by ANOVA with **Gender** (female and male participant) as between-subject variable and **Movement** (Approaching and Withdrawal) as within-subject factors.

To study the relationship between preferred interpersonal distance and physiological measure, first, the best-fitting linear equations, relating SCR data points to spatial positions in the approach condition, were computed for each participant. Then, the resultant slope parameters, indicating the rate at which SCR changed (i.e., steepness) as the confederate approached the participant, were correlated with participants preferred interpersonal distance as assessed in the stop-distance task.
